# Editorial: Genome-based nutrition strategies for preventing diet-related chronic diseases: where genes, diet, and food culture meet

**DOI:** 10.3389/fnut.2024.1441685

**Published:** 2024-06-24

**Authors:** Arturo Panduro, Claudia Ojeda-Granados, Omar Ramos-Lopez, Sonia Roman

**Affiliations:** ^1^Department of Genomic Medicine in Hepatology, Civil Hospital of Guadalajara, Guadalajara, Jalisco, Mexico; ^2^Health Sciences Center, University of Guadalajara, Guadalajara, Jalisco, Mexico; ^3^Department of Medical and Surgical Sciences and Advanced Technologies “GF Ingrassia, ” University of Catania, Catania, Italy; ^4^Medicine and Psychology School, Autonomous University of Baja California, Tijuana, Baja California, Mexico

**Keywords:** personalized nutrition, personalized medicine, chronic diseases, gene polymorphisms, gene-environmental mismatch, ancestry, lifestyle factors, omic sciences

## Introduction

Weight gain is often the initial trigger for metabolic abnormalities and chronic diseases such as obesity, type 2 diabetes, cardiovascular disease, metabolic-associated steatotic liver disease, and many malignancies worldwide. These emerging diseases have spread across the global population, causing significant morbidity and mortality (1, 2). In the past, it was common to attribute the etiology of these disorders to “genetics” or label them as “multifactorial” when a definitive explanation was lacking. However, since the first human genome blueprint was released nearly 25 years ago, it has been a pivotal moment, signaling the transition from the pre-genomic to the genomic and post-genomic era^1^ (3). This transition has led us to the development of the multi-omic sciences, an interdisciplinary field that provides a comprehensive and holistic understanding of gene structure, gene expression mechanisms, and how environmental interactions affect them (4–6). It is now clear that attributing most prevalent chronic diseases solely to genetics or the environment is no longer accurate (7). The interaction of these factors plays a significant role in maintaining our health or leading to disease, and they can be identified more precisely (8).

Almost 20 years ago, we recognized physical activity, emotions, and diet as the key lifestyle factors influencing our genes (9, 10). However, we also acknowledged the genetic heterogeneity that any region or population would exhibit due to local adaptation.

Thus, regional studies are critical, focusing on the prevalence of such adaptive genes and assessing the influence of lifestyle changes at the population level (11, 12). Despite global modifications in lifestyle conditions, the human genome remains tailored to past environments. This leads to a key concept in the era of personalized medicine and nutrition- the gene-environmental mismatch (13). This mismatch underlines the importance of tailoring preventive strategies depending on the target population's genetic composition and environmental context rather than relying on general health guidelines to regain health.

## A new era of genome-based nutrition strategies

This Research Topic on “*Genome-based nutrition strategies for preventing diet-related chronic diseases*” aimed to provide readers with a compilation of studies on the role of the genetic background of individuals and populations in diseases such as obesity and associated comorbidities as well as on prevention and intervention strategies that rely on evidence of the gene-environment mismatch or imbalance that contributes to the development of these diseases, to promote the consumption of genetically and culturally respectful and sustainable diets.

Roman et al.'s perspective on the end of the one-diet-fits-all paradigm underlines the necessity for personalized nutrition strategies based on the target population's genetic profile, regional foods, and food culture. Mexico, a country with admixed ancestry, heterogenic diet-related risk alleles, and a negative nutrition transition, is affected by the one-diet-fits-all regimen. It also discusses how the Genomex diet, a nutrigenetic strategy, can prevent and correct metabolic abnormalities among the population.

However, how can we start using a Personalized Nutrition approach to prevent chronic diseases? Panduro et al. suggest that understanding the impact of genes and the environment at the level of each geographical region is the first step toward developing prevention measures tailored to each population and individual. In their review, the authors define key factors that should be considered to prevent and treat the main chronic diseases, particularly liver diseases, through a Personalized Medicine and Nutrition (PerMed-Nut) model. In addition, they stress the need for training medical students and professionals in genomics education to guarantee the successful implementation of such strategies.

Genetic backgrounds and environmental contexts can affect major chronic metabolic disease predispositions and frequency patterns and must be considered in personalized and regionalized nutritional approaches. In their mini-review, Zambrano et al. discuss the connection between nutrition and genetic variants that cause hypertension and how these interactions affect groups differently in regions of Latin America. In contrast, Ojeda-Granados et al. discuss the epidemiology of cardiovascular diseases, particularly in Italy, where they are the leading cause of death, and the genetic, lifestyle/behavioral, and metabolic factors that increase risk.

Following this path, Nacis et al. confirm that to achieve the goals of a Precision Nutrition approach, gene-based nutrition, and lifestyle recommendations need to be integrated into clinical practice to address metabolic variability between individuals. Therefore, the authors present their MyGeneMyDiet^®^ study protocol, which aims to evaluate the efficacy of providing nutrition and lifestyle recommendations based on individual genotypes of single nucleotide polymorphisms influencing body weight, calorie, and fat intake in weight management among adults.

Mera-Charria et al. examined the connection of numerous obesity-related gene polymorphisms with body mass index and weight loss therapy response in a multiethnic cohort to understand obesity and prevent its comorbidities. The authors' work shed light on hereditary factors affecting weight and weight loss treatment responsiveness. Rivera-Iñiguez et al. examined the link between gene polymorphisms regulating energy balance and food reward and appetitive traits in young Mexican subjects, particularly with disordered eating patterns and obesity. They found that genetic variables may increase hedonic food consumption and decrease satiety regulation, causing weight gain. The authors present more evidence for individualized nutrigenetic techniques to combat disordered eating.

To add evidence on how particular dietary patterns contribute to obesity and metabolic complications, Zhang et al. employed 16S rRNA sequencing and untargeted metabolomic analysis in a rat model to examine how a high-fat diet affects gut microbiota and metabolism to demonstrate how certain diets cause obesity and metabolic issues. Long-term high-fat diet consumption in rats caused dyslipidemia, intestinal bacteria changes, and plasma metabolism changes.

Traditional diets rich in fruit, vegetables, grains, and essential fatty acids improve chronic inflammatory profiles, which are prominent in diet-related chronic illnesses. Therefore, in the last study of our Research Topic, Díez-Sainz et al. explored the impact of vegetable-rich diets on inflammation and the identification of bioactive molecules and their mechanisms of action. They focused on the modulation of human pro-inflammatory genes by edible plant-derived microRNAs. Their findings revealed that particular microRNAs can promote an anti-inflammatory gene expression profile in human macrophages *in vitro* and that diet may increase bioavailability but eventually limit it at the intestine level.

## Final considerations

The contributions in this Research Topic are evidence of the myriad of combined multi-omic sciences approaches and qualitative methodologies needed to provide personalized medicine or nutrition strategies based on the specific genetic and cultural aspects of health. Future studies worldwide will continue to provide regionalized data that can guide the implementation of such strategies. It will also require genomic education and training of past and future generations of scholars in the healthcare field to interpret genomic data and apply preventive measures to avoid chronic diseases among the target populations.

## Author contributions

AP: Writing – review & editing, Writing – original draft, Conceptualization. CO-G: Writing – review & editing, Writing – original draft. OR-L: Writing – review & editing. SR: Writing – review & editing, Conceptualization.

## Footnotes

^1^The Human Genome Project. Available online at: https://www.genome.gov/human-genome-project (accessed May 28, 2024).
